# Supplementary material for: Microbial community dissimilarity for source tracking with application in forensic studies
Source: PLoS One. 2020 Jul 23;15(7):e0236082. doi: 10.1371/journal.pone.0236082 (PMC7377425; doi:10.1371/journal.pone.0236082)
Supplement: S1 File — (DOCX) [file pone.0236082.s001.docx]

**Supplementary file**

**Appendix A: Benchmark Data Statistics**

**Table S1. Unique OTUs and reads in unique OTUs by source**

| Source | Unique OTUs | %Unique OTUs | Unique Reads | %Unique Reads |
| --- | --- | --- | --- | --- |
| A | 94.00 | 28.75 | 121.00 | 4.84 |
| B | 118.00 | 33.62 | 206.00 | 8.24 |
| C | 94.00 | 41.96 | 210.00 | 8.40 |
| D | 93.00 | 36.61 | 219.00 | 8.76 |
| E | 108.00 | 40.15 | 366.00 | 14.64 |
| F | 140.00 | 42.94 | 287.00 | 11.48 |
| G | 104.00 | 38.52 | 156.00 | 6.24 |
| H | 180.00 | 40.18 | 212.00 | 8.48 |
| I | 188.00 | 39.58 | 249.00 | 9.96 |
| J | 175.00 | 49.02 | 355.00 | 14.20 |

**Table S2. Percentage of shared OTUs for individual sources and evidence ( mixture of three samples)**

|  | A | B | C | D | E | F | G | H | I | J |
| --- | --- | --- | --- | --- | --- | --- | --- | --- | --- | --- |
| A | 100.00 | 43.12 | 20.80 | 24.46 | 23.85 | 33.33 | 27.52 | 41.28 | 41.59 | 29.97 |
| B | 40.17 | 100.00 | 18.52 | 23.65 | 22.22 | 27.92 | 25.93 | 37.89 | 41.31 | 29.06 |
| C | 30.36 | 29.02 | 100.00 | 26.79 | 29.46 | 25.45 | 17.86 | 30.80 | 33.48 | 27.68 |
| D | 31.50 | 32.68 | 23.62 | 100.00 | 32.68 | 28.35 | 23.62 | 36.61 | 39.76 | 31.10 |
| E | 29.00 | 29.00 | 24.54 | 30.86 | 100.00 | 23.42 | 18.22 | 31.23 | 37.55 | 24.91 |
| F | 33.44 | 30.06 | 17.48 | 22.09 | 19.33 | 100.00 | 25.15 | 30.37 | 30.98 | 24.23 |
| G | 33.33 | 33.70 | 14.81 | 22.22 | 18.15 | 30.37 | 100.00 | 35.93 | 38.89 | 25.56 |
| H | 30.13 | 29.69 | 15.40 | 20.76 | 18.75 | 22.10 | 21.65 | 100.00 | 38.39 | 22.10 |
| I | 28.63 | 30.53 | 15.79 | 21.26 | 21.26 | 21.26 | 22.11 | 36.21 | 100.00 | 24.63 |
| J | 27.45 | 28.57 | 17.37 | 22.13 | 18.77 | 22.13 | 19.33 | 27.73 | 32.77 | 100.00 |
| .6B:.3D:.1G | 30.74 | 57.87 | 15.31 | 37.28 | 22.39 | 24.50 | 29.16 | 32.63 | 34.99 | 24.74 |
| .5B:.3D:.2G | 31.50 | 53.50 | 15.94 | 37.37 | 21.79 | 25.91 | 33.62 | 34.63 | 38.38 | 25.67 |
| .4B:.3D:.3G | 30.97 | 49.42 | 14.85 | 38.50 | 20.05 | 23.31 | 35.79 | 33.00 | 35.61 | 24.36 |
| .33B:.33D:.33G | 31.89 | 47.88 | 16.62 | 39.70 | 22.44 | 26.26 | 37.41 | 33.75 | 34.54 | 25.28 |

**Appendix B: Two-Source Mixtures**


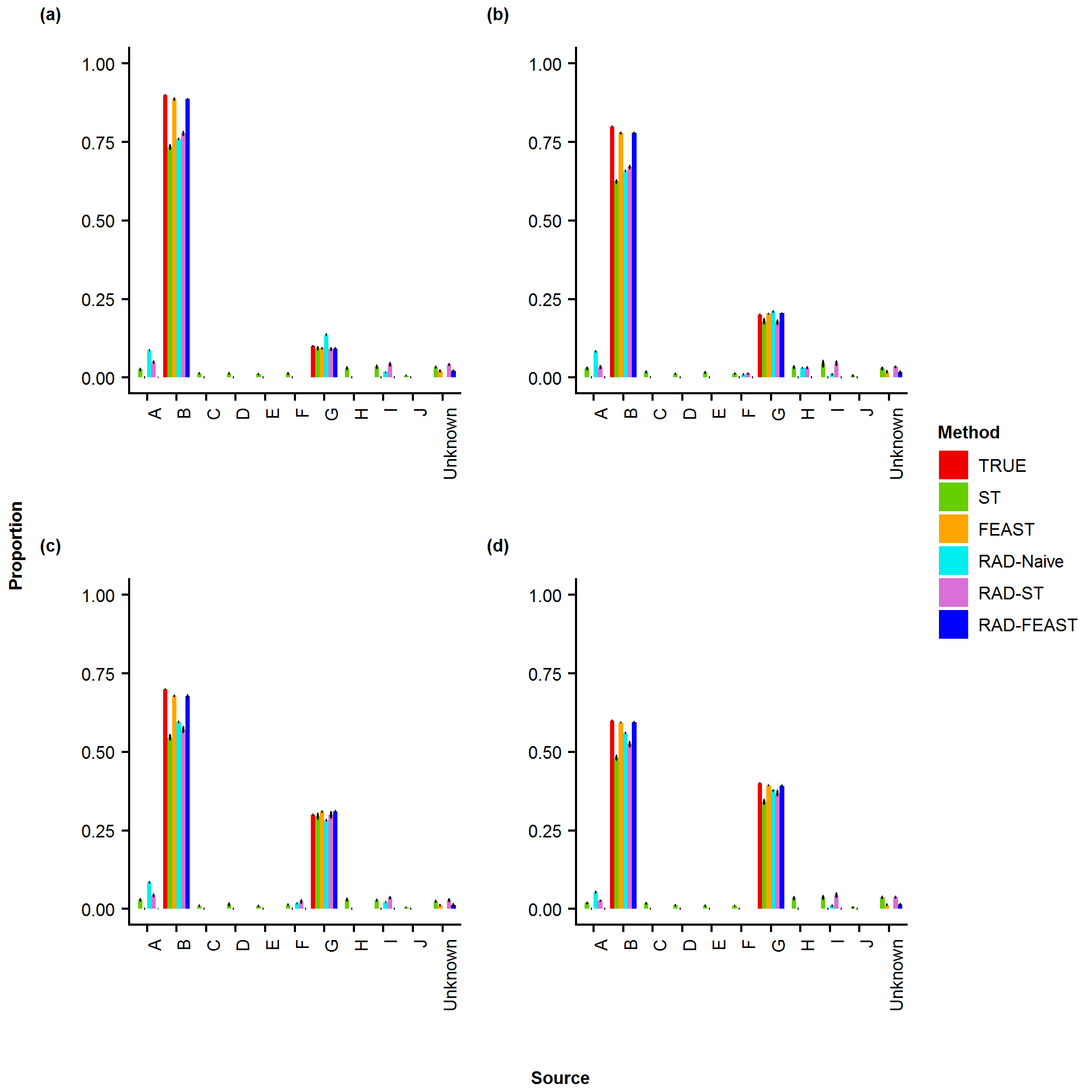


**Fig S1. Proportion estimates for two-source mixtures.** Comparison of true mixture proportion with estimated proportions of for various mixtures: (a) 90%-10%, (b) 80% - 20%, (c) 70% - 30%, (d) 60% - 40%.


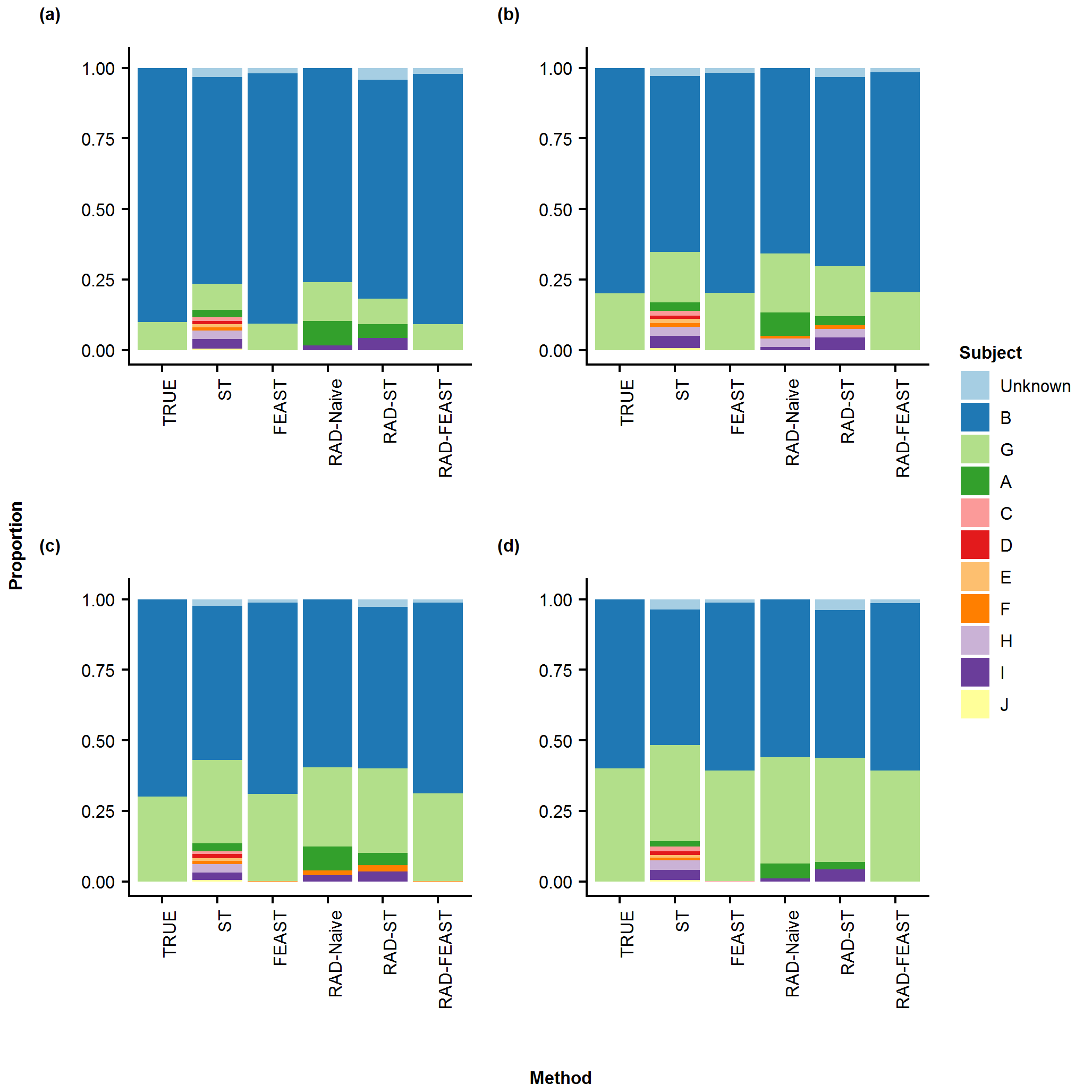


**Fig S2. Stacked-bar plots for two-source mixtures.** Comparison of true mixture proportion with mean proportions of various mixture settings: (a) 90%-10%, (b) 80% - 20%, (c) 70% - 30%, (d) 60% - 40%.


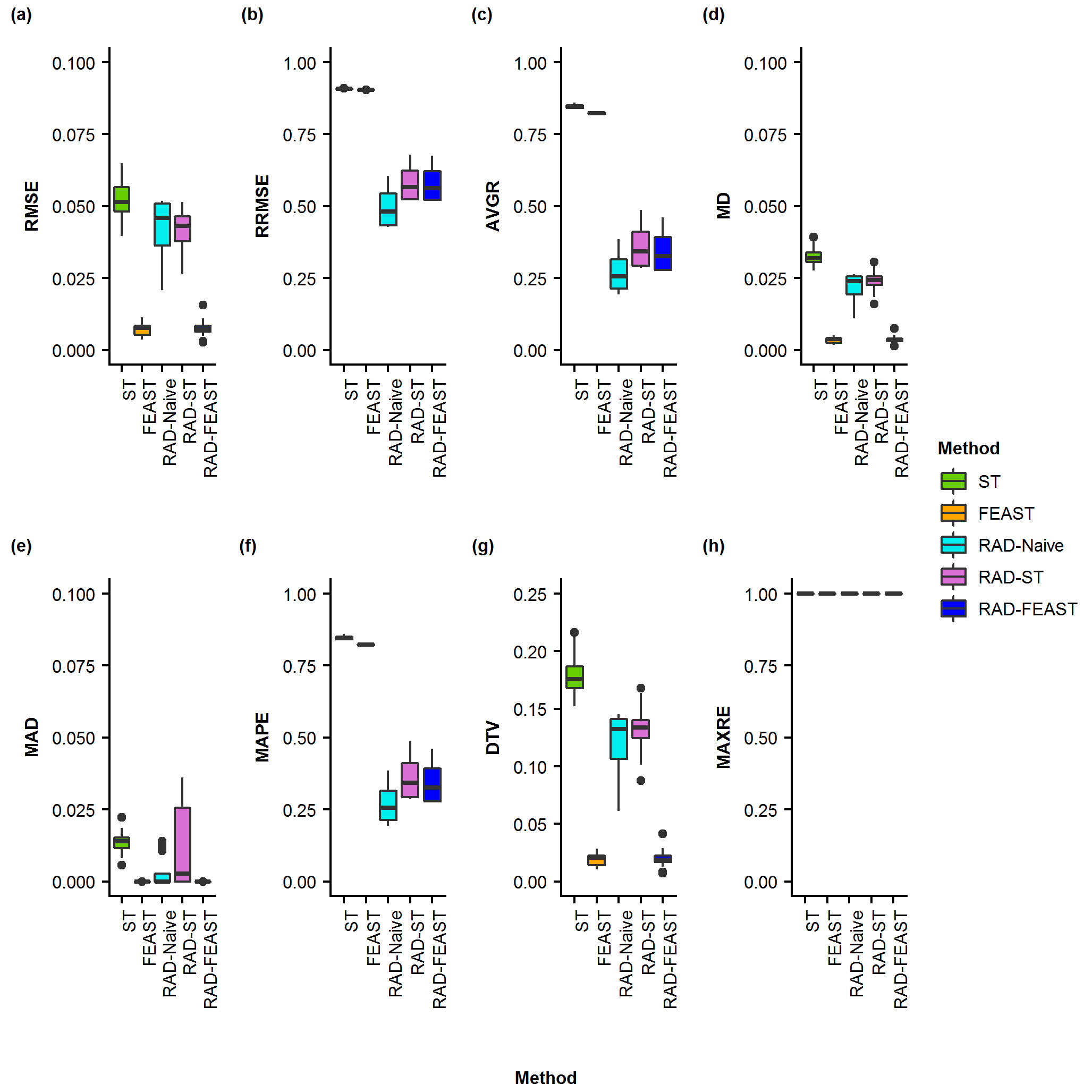


**Fig S3. Proportion errors for two-source mixtures.** Comparison of error measurements for SourceTracker (ST), FEAST, RAD-Naive, RAD-ST, and RAD-FEAST of for various mixtures: (a) Root Mean Square Error, (b) Relative Root Mean Square Error, (c) Average Residual Error, (d) Mean Difference, (e) Median Absolute Deviation, (f) Mean Absolute Percentage Error, (g) Total Variation Distance, and (h) Maximum Residual Error.


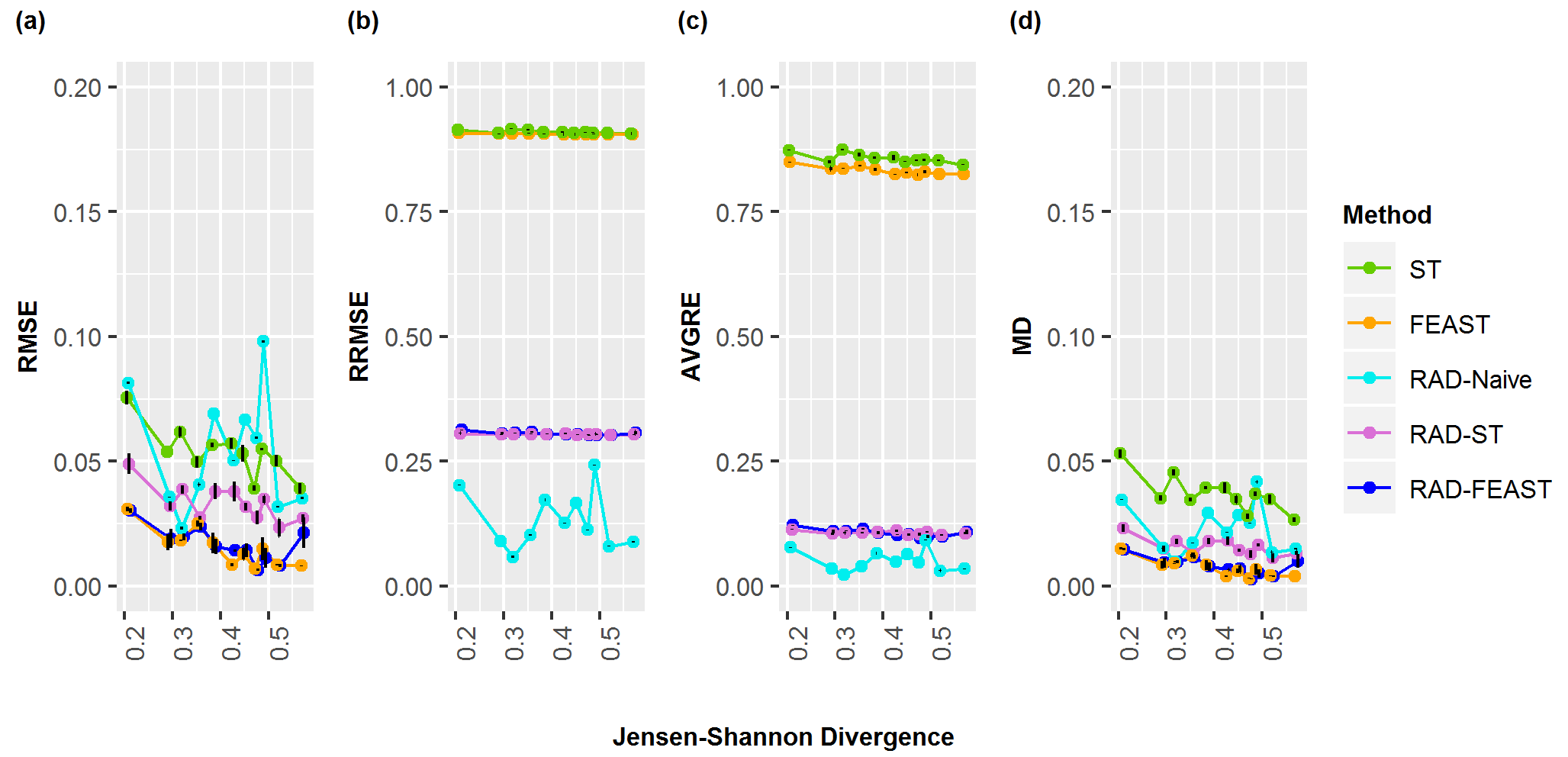


**Fig. S4. Proportion errors for two-source mixtures by divergence.** Comparison of error measurements for SourceTracker (ST), FEAST, RAD-Naive, RAD-ST, and RAD-FEAST for 70% - 30% evidence mixtures with varying mixture sources representing differing levels of Jensen-Shannon divergence: (a) Root Mean Square Error, (b) Relative Root Mean Square Error, (c) Average Residual Error, and (d) Mean Difference.

**Appendix C: Four-Source Mixtures**

**
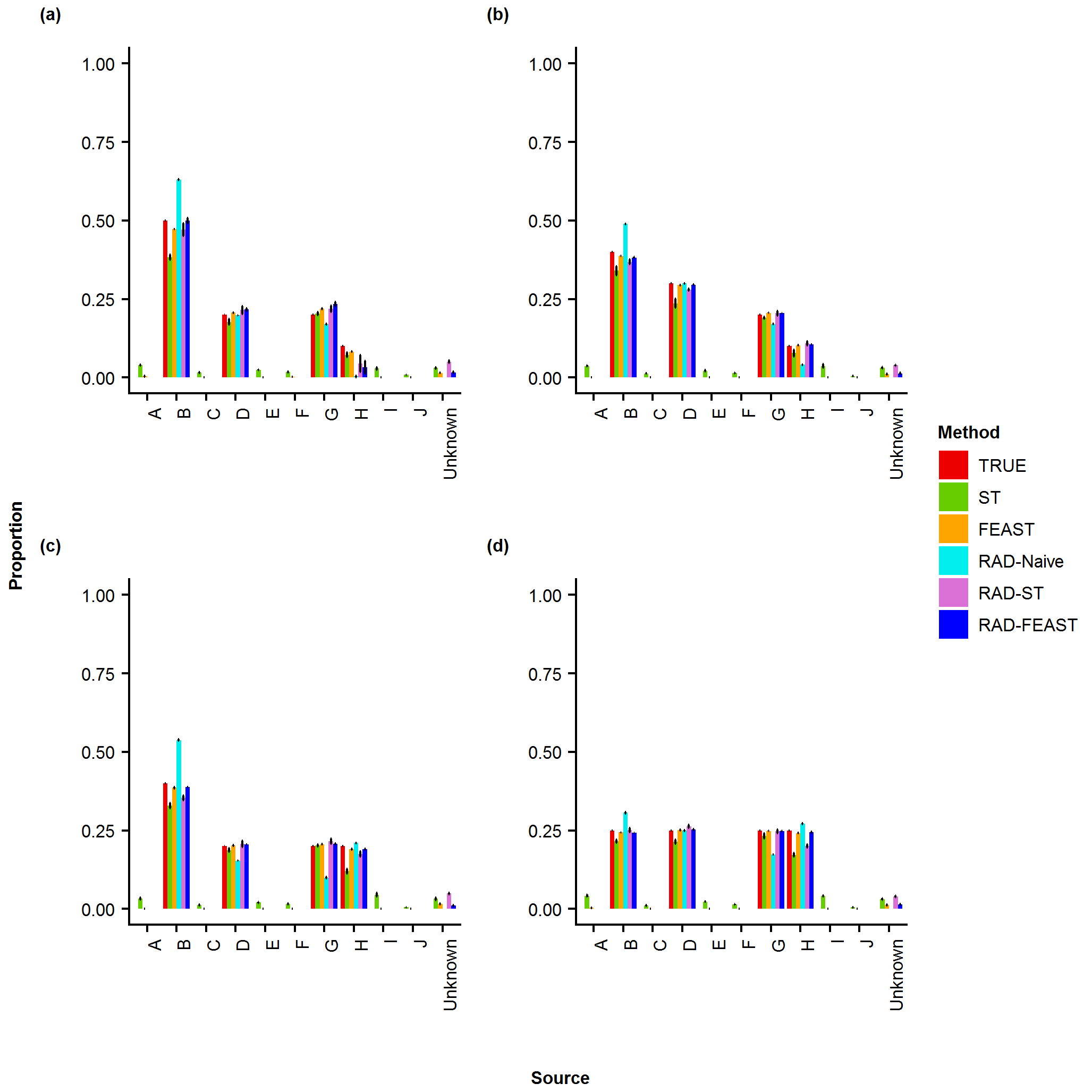
**

**Fig S5. Proportion estimates for four-source mixtures.** Comparison of true mixture proportion with estimated proportions of for various mixtures: (a) 50%-20%-20%-10%, (b) 40%-30%-20%-10%, (c) 40%-20%-20%-20%, (d) 25%- 25%-25%-25%.


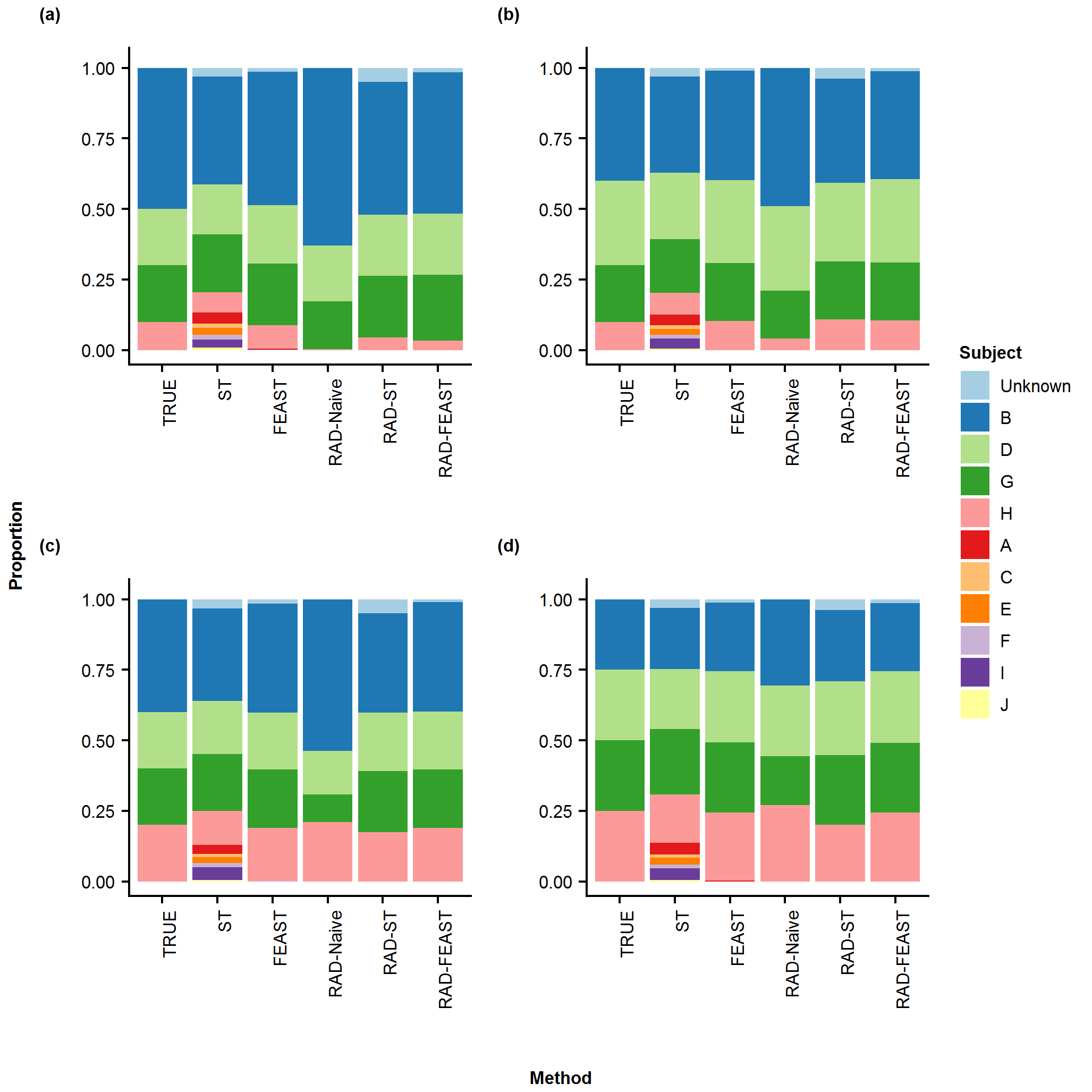


**Fig S6. Stacked bar plots for four-source mixtures.** Comparison of true mixture proportion with mean proportions of various mixture settings: (a) 50%-20%-20%-10%, (b) 40%-30%-20%-10%, (c) 40%-20%-20%-20%, (d) 25%- 25%-25%-25%.


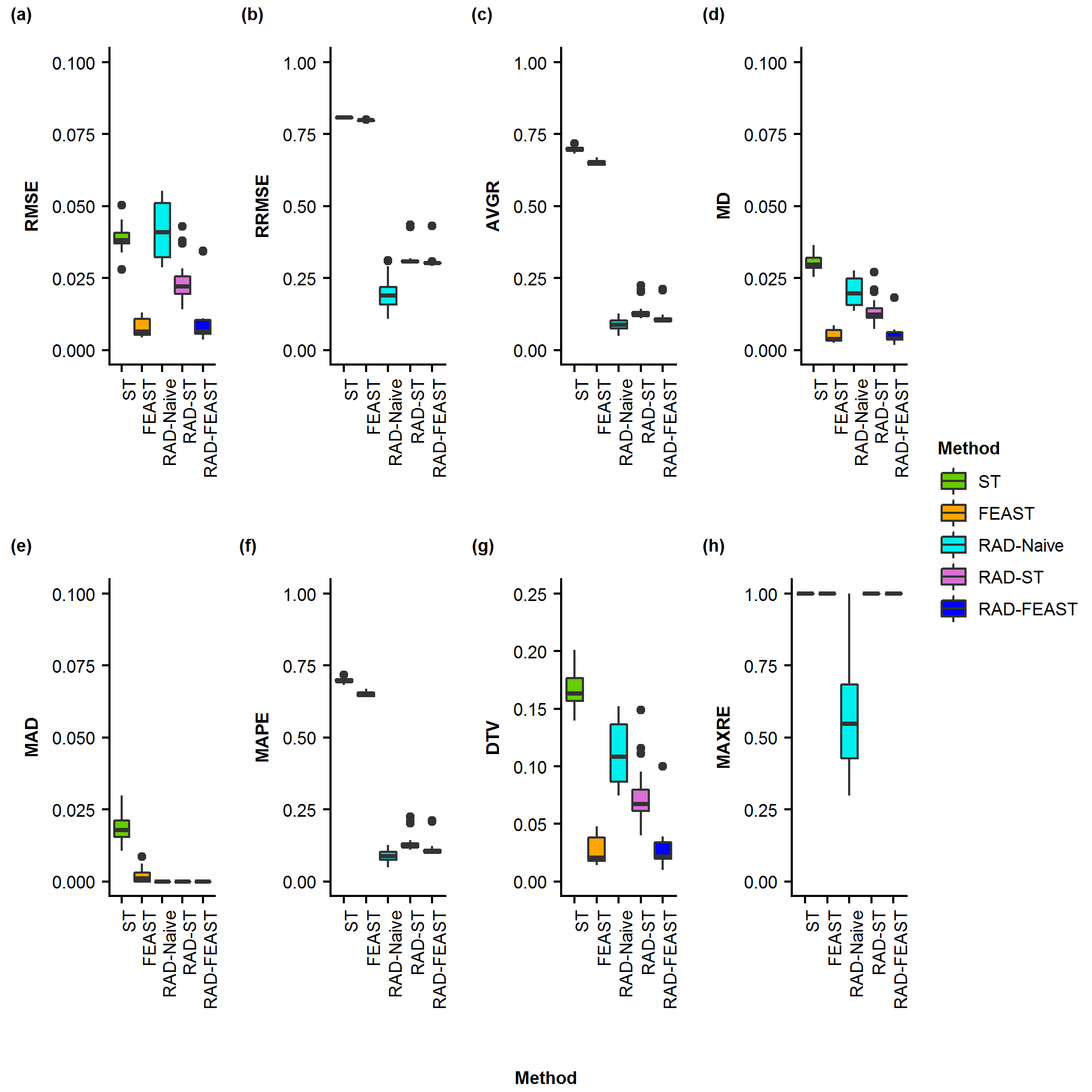


**Fig S7. Proportion errors for four-source mixtures.** Comparison of error measurements for SourceTracker (ST), FEAST, RAD-Naive, RAD-ST, and RAD-FEAST of for various mixtures: (a) Root Mean Square Error, (b) Relative Root Mean Square Error, (c) Average Residual Error, (d) Mean Difference, (e) Median Absolute Deviation, (f) Mean Absolute Percentage Error, (g) Total Variation Distance, and (h) Maximum Residual Error.

**Appendix D: Three Source Mixtures for different days**

**
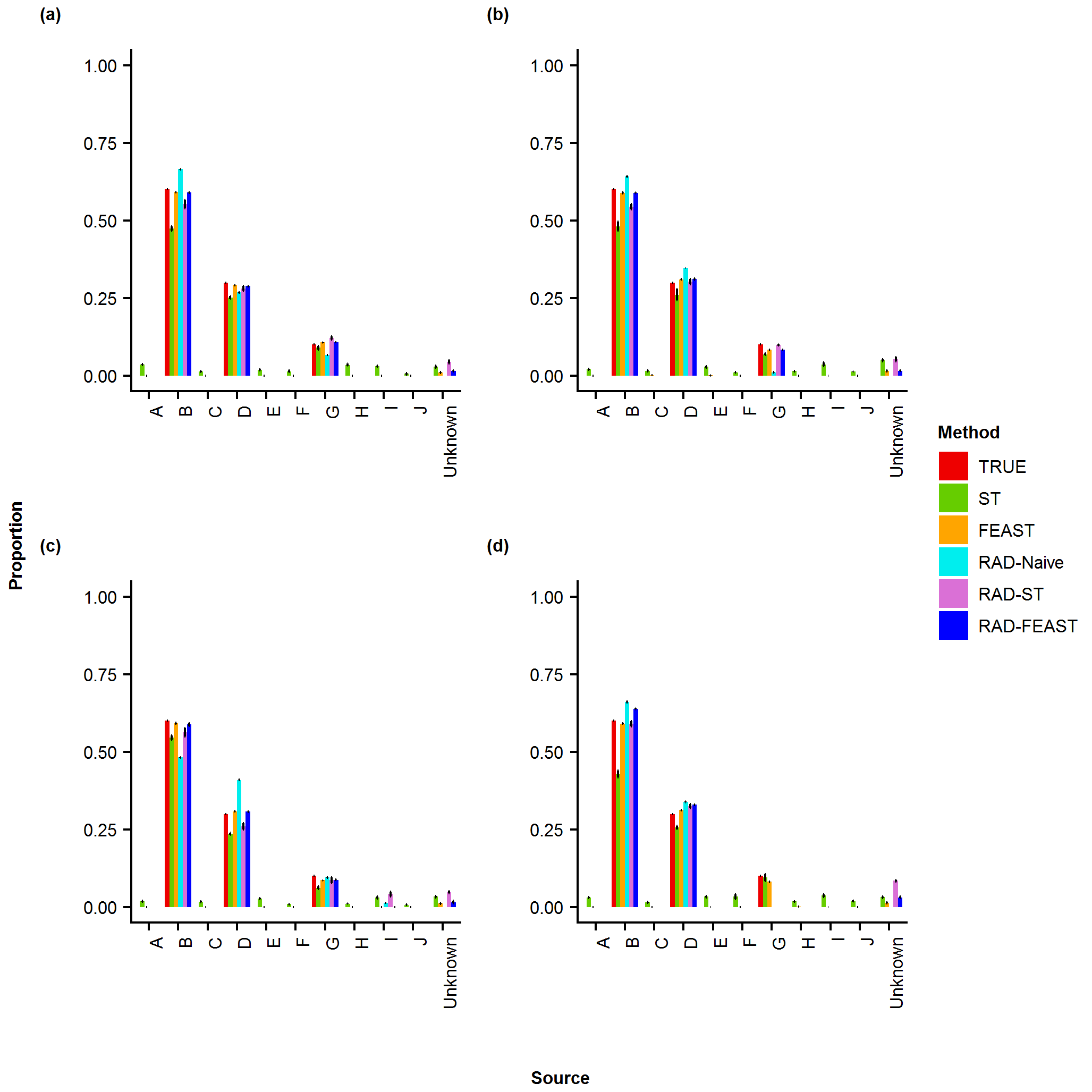
**

**Fig S8. Proportion estimates for different days.** Comparison of true mixture proportion with estimated proportions for a 60%-30%-10% mixture on Day 1 using samples from a later day: (a) Day 2, (b) Day 3, (c) Day 4, (d) Day 5.


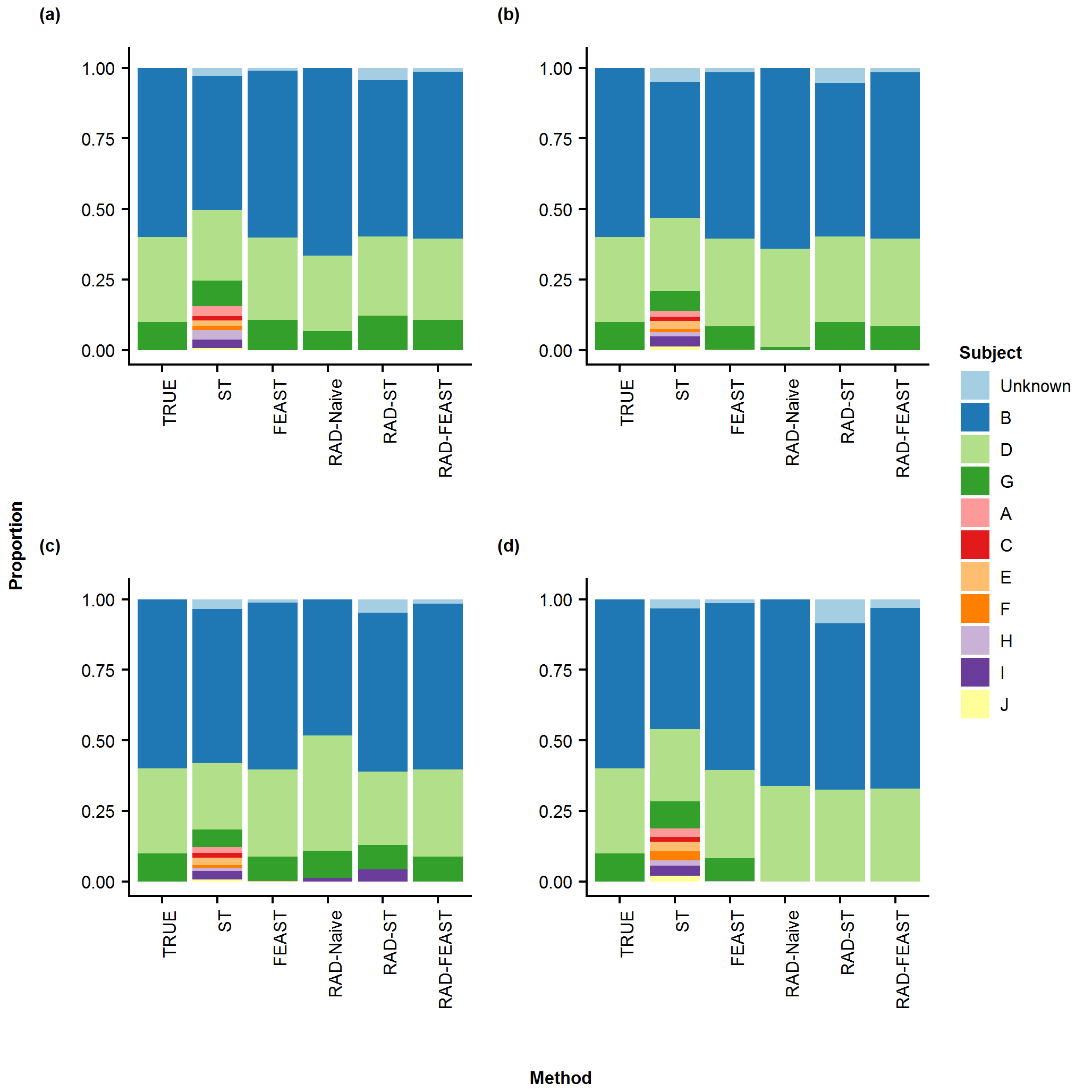


**Fig S9. Stacked-bar plots for different days.** Comparison of true mixture proportion with mean proportions for a 60%-30%-10% mixture on Day 1 using samples from a later day: (a) Day 2, (b) Day 3, (c) Day 4, (d) Day 5.


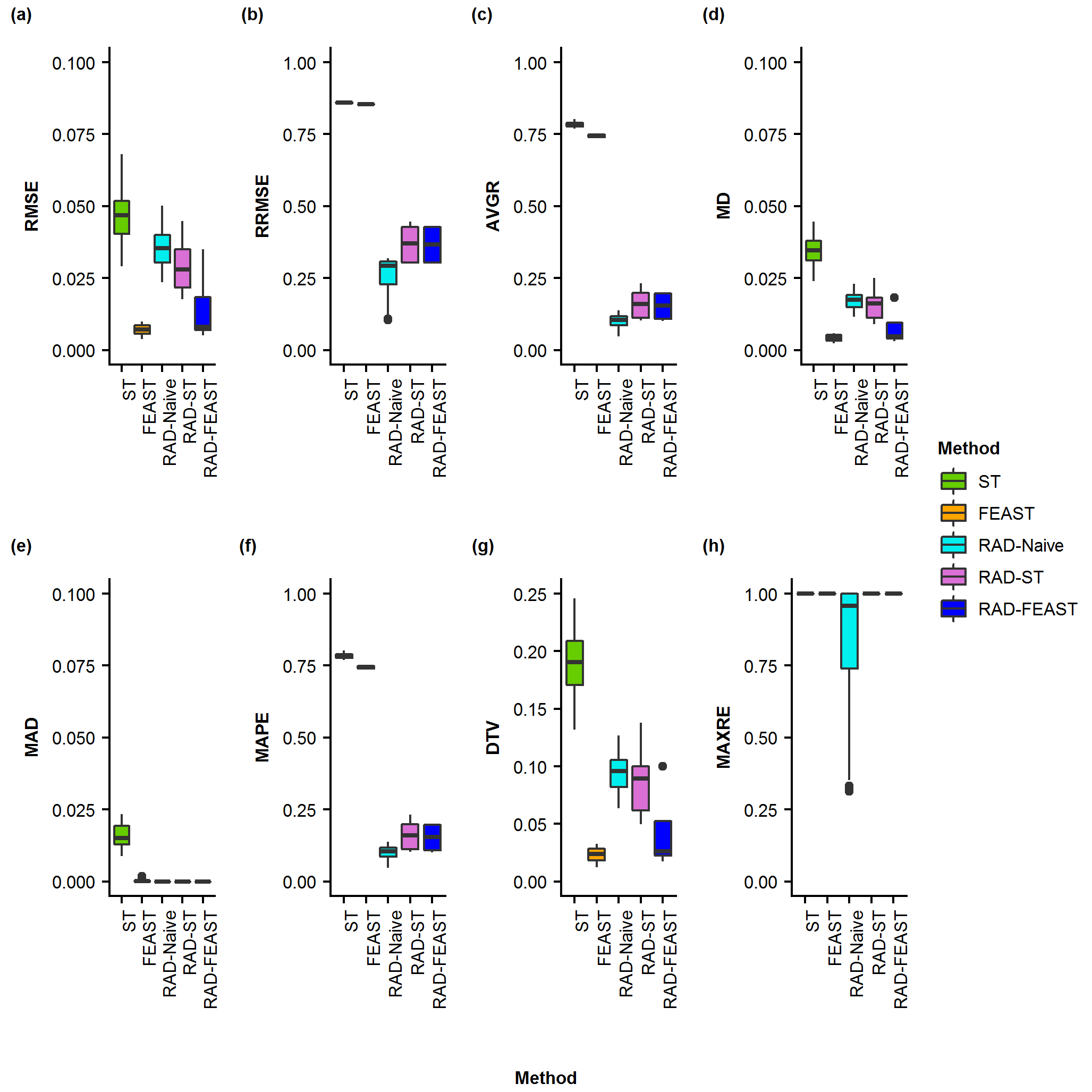


**Fig S10. Proportion errors for two-source mixtures for day 2~5 (cumulatively).** Comparison of error measurements for SourceTracker (ST), FEAST, RAD-Naive, RAD-ST, and RAD-FEAST for a 60%-30%-10% mixture on Day 1 using samples from the following four days: (a) Root Mean Square Error, (b) Relative Root Mean Square Error, (c) Average Residual Error, (d) Mean Difference, (e) Median Absolute Deviation, (f) Mean Absolute Percentage Error, (g) Total Variation Distance, and (h) Maximum Residual Error.

**Appendix E: Additional Three Source Mixtures (Evidence Mixture of Sources A, B, and F)**


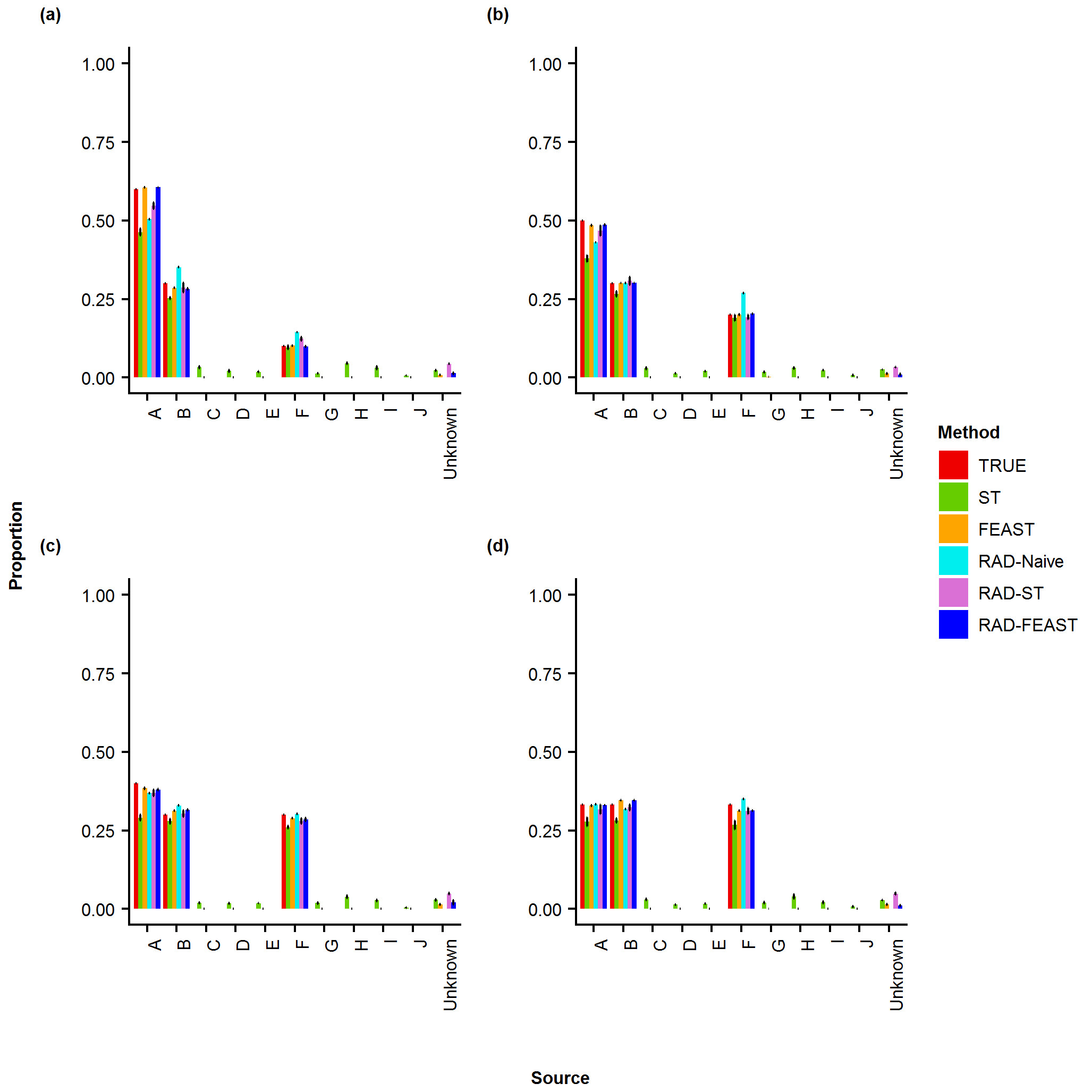


**Fig S11. Proportion estimates for three-source mixtures composed of sources A, B, and F.** Comparison of true mixture proportion with estimated proportions of various mixture settings: (a) 60% - 30% - 10%, (b) 50% - 30% - 20%, (c) 40% - 30% - 30%, and (d) 33% - 33% - 33%.


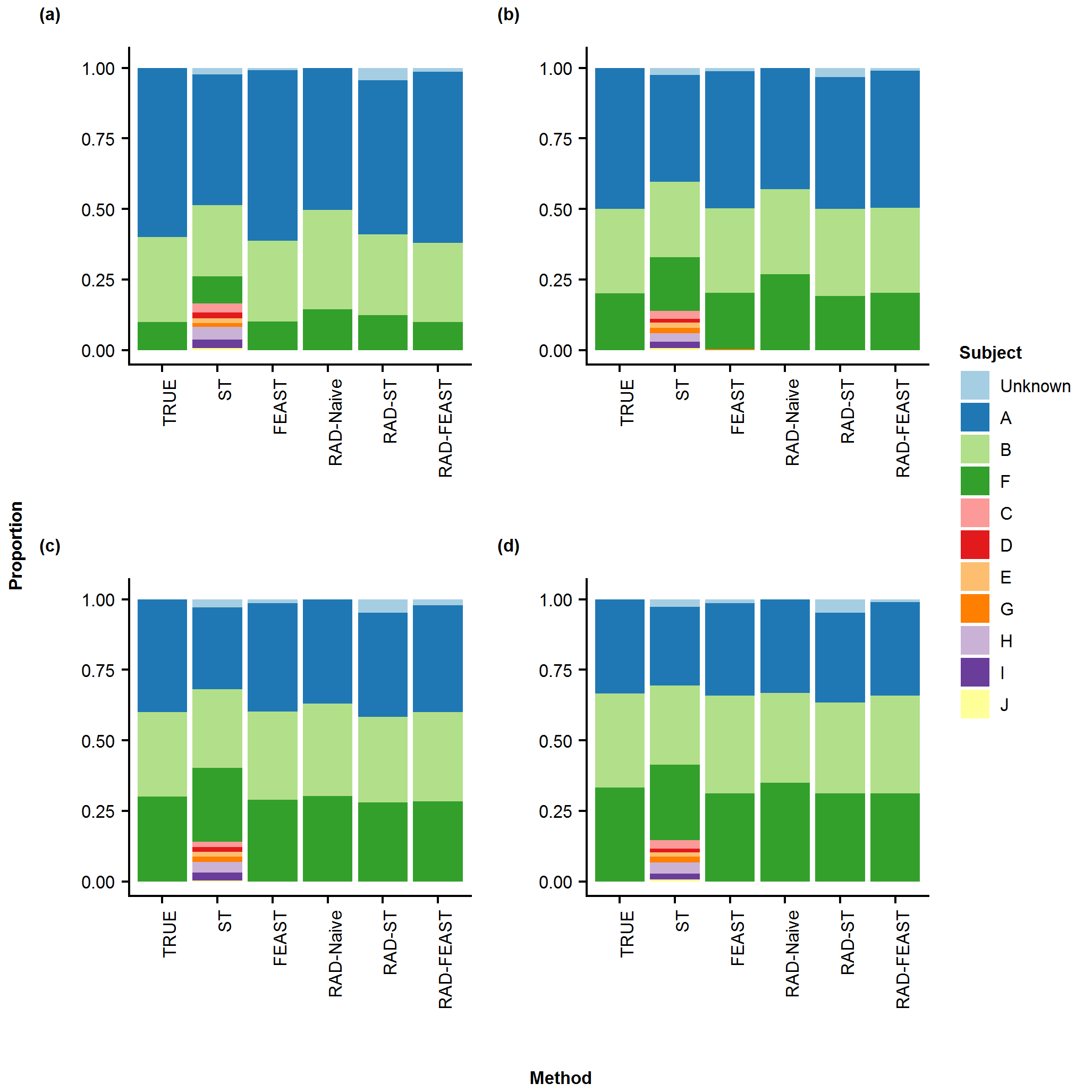


**Fig S12. Stacked-bar plots for three-source mixtures composed of sources A, B, and F.** Comparison of true mixture proportion with mean proportions of various mixture settings: (a) 60% - 30% - 10%, (b) 50% - 30% - 20%, (c) 40% - 30% - 30%, and (d) 33% - 33% - 33%.


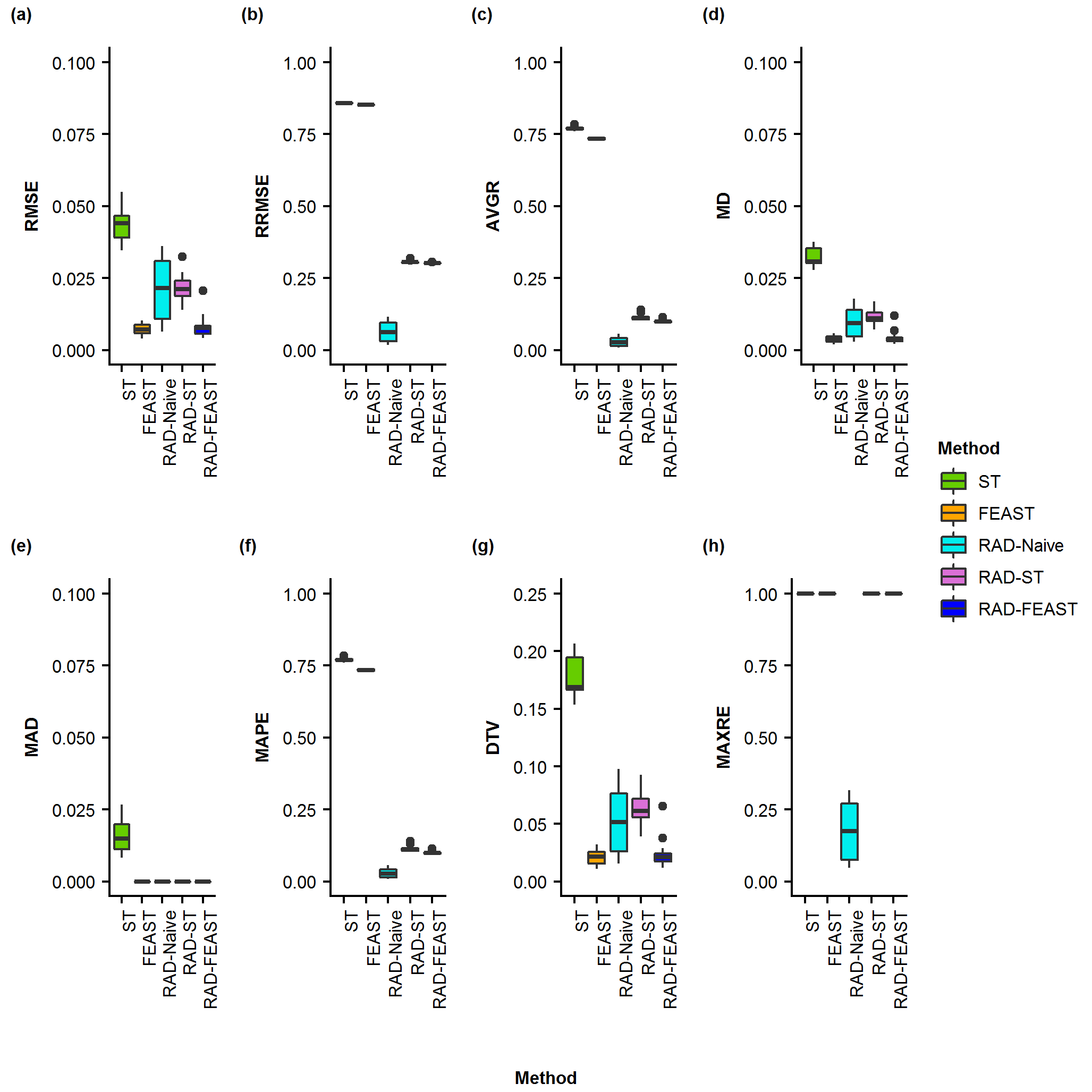


**Fig S13. Proportion errors for three-source mixtures composed of sources A, B, and F.** Comparison of error measurements for SourceTracker (ST), FEAST, RAD-Naive, RAD-ST, and RAD-FEAST across various mixture settings: (a) Root Mean Square Error, (b) Relative Root Mean Square Error, (c) Average Residual Error, (d) Mean Difference, (e) Mean Absolute Deviation, (f) Mean Absolute Percentage Error, (g) Total Variation Distance, and (h) Maximum Residual Error.
