# Supplementary material for: Microbial community dissimilarity for source tracking with application in forensic studies
Source: PLoS One. 2020 Jul 23;15(7):e0236082. doi: 10.1371/journal.pone.0236082 (PMC7377425; doi:10.1371/journal.pone.0236082)
Supplement: Attachment — Submitted filename: PONE-D-19-24969_review.pdf [file pone.0236082.s002.pdf]

This manuscript describes the use of proportional distance metrics to disentangle mixtures of microbial communities contributing samples. This is a reasonable approach since many previous studies have considered how using proportional data metrics might be more representative compared to absolute measures of error. As with any study of this kind, analysis of this approach will heavily depend on the benchmark dataset and methodology used.

The benchmark dataset used is derived from OTU tables of 16S sequencing from Lax et al, which is rarefied to 2500 counts per sample. Rarefaction is normally used for methods which cannot handle proportional data, but in this case, the proposed method RAD should not be harmed by differing sequencing depths per sample. In this case, it is ideal to fully utilize the data instead of rarefied counts. It would be interesting to see if RAD performs better with the full unrarefied counts, but I understand this analysis might be too difficult to perform because the full pipeline of generating the OTU counts might be unclear. Acknowledgement and discussion of whether rarefaction is appropriate would be a reasonable substitute if OTUs cannot be recomputed.

The main benchmarking dataset may be a favorable benchmark dataset because of the limited number of sources for each mixture (two or three). It would be greatly informative to the reader if more statistics about the benchmarking datasets were available, such as the number of unique OTUs in the source and mixtures, the original Aitchison distances between these sources, the sparseness of pairwise OTU counts, conventional distance metrics like L2, and any other metrics the author may find informative to understand the benchmark dataset.

The results go on to focus on how RAD might improve ST and FEAST via source removal when considering two and three source mixtures. How does RAD compare to simplistic post-processing source removal approaches? One very basic approach might be to simply remove sources from ST/FEAST output if their estimated proportion is <X%, set to a relatively small number such as X=1%, then redistribute the removed density to the leftover sources. This adds a user-selected threshold X, which RAD doesn't require. RAD may still be useful as a parameterless approach. However, it would be greatly informative to understand how RAD compares to basic thresholding.

Minor comments:
There does not appear to be a file representing the summary data used to generate the Figures publically available. There is also no source code that I could find at: https://cals.arizona.edu/~anling/software/RAD/

In part 2.1, hierarchical clustering is mentioned but it is unclear whether it was used for the simulation analysis.

In part 2.2.2 with ST, it is not clear what the hyperparameters alpha and beta are set to after parameter tuning, or how they were tuned.
