# Supplementary material for: Microbial community dissimilarity for source tracking with application in forensic studies
Source: PLoS One. 2020 Jul 23;15(7):e0236082. doi: 10.1371/journal.pone.0236082 (PMC7377425; doi:10.1371/journal.pone.0236082)
Supplement: Attachment — Submitted filename: PONE_Response_to_Reviewers_final.docx [file pone.0236082.s003.docx]

**Response to Reviewers (Manuscript ID:** **PONE-D-19-24969)**

**Reviewer 1**

1. The benchmark dataset used is derived from OTU tables of 16S sequencing from Lax et al, which is rarefied to 2500 counts per sample. Rarefaction is normally used for methods which cannot handle proportional data, but in this case, the proposed method RAD should not be harmed by differing sequencing depths per sample. In this case, it is ideal to fully utilize the data instead of rarefied counts. It would be interesting to see if RAD performs better with the full unrarefied counts, but I understand this analysis might be too difficult to perform because the full pipeline of generating the OTU counts might be unclear. Acknowledgement and discussion of whether rarefaction is appropriate would be a reasonable substitute if OTUs cannot be recomputed.

Thank you for your input! While Aitchison distance does handle proportional data, for microbiome data, we still expect minor changes in mixtures with unequal library size. As the library size decreases, the number of some features may drop to zero by under-sampling. We have investigated the behavior by simulating samples with varying total counts (i.e., library size). The graphic below shows the behavior of Relative Aitchison Difference (RAD) of the sample with the largest library size to the evidence sample as the ratio between library sizes of two sources increases (from 1:1 to 1:8) using 100 replicates. We can observe some changes in the RAD values, but the testing result of whether the source is significant (i.e., RAD is greater than zero) stays consistent.

2. The main benchmarking dataset may be a favorable benchmark dataset because of the limited number of sources for each mixture (two or three). It would be greatly informative to the reader if more statistics about the benchmarking datasets were available, such as the number of unique OTUs in the source and mixtures, the original Aitchison distances between these sources, the sparseness of pairwise OTU counts, conventional distance metrics like L2, and any other metrics the author may find informative to understand the benchmark dataset.

Thank you for your comment. The choice of mixture was chosen to reflect a difficult scenario where only one person/source from three families contributes to the evidence sample meaning the source must be identified by individual rather than by family. Below are some metrics of the dataset including the number of unique OTUs in each sample, the percentage of OTUs shared between samples, the percentage of total counts within shared OTUs, and the Aitchison distance between sample (Euclidean distance metrics are inappropriate for ecological comparisons since do not compare within-species). From the tables below we can infer that the selected samples in our original simulation settings (highlighted) are good representations as they are not quite distinct from others.

**Benchmark dataset characteristics:**

Table of unique OTUs and uniqure reads for each sample/source.

| Sample | Unique.OTUs | %Unique.OTUs | Unique.Reads | %Unique.Reads |
| --- | --- | --- | --- | --- |
| A | 94.00 | 28.75 | 121.00 | 4.84 |
| B | 118.00 | 33.62 | 206.00 | 8.24 |
| C | 94.00 | 41.96 | 210.00 | 8.40 |
| D | 93.00 | 36.61 | 219.00 | 8.76 |
| E | 108.00 | 40.15 | 366.00 | 14.64 |
| F | 140.00 | 42.94 | 287.00 | 11.48 |
| G | 104.00 | 38.52 | 156.00 | 6.24 |
| H | 180.00 | 40.18 | 212.00 | 8.48 |
| I | 188.00 | 39.58 | 249.00 | 9.96 |
| J | 175.00 | 49.02 | 355.00 | 14.20 |

Table of shared OTUs (%).

|  | A | B | C | D | E | F | G | H | I | J |
| --- | --- | --- | --- | --- | --- | --- | --- | --- | --- | --- |
| A | 100.0 | 43.12 | 20.80 | 24.46 | 23.85 | 33.33 | 27.52 | 41.28 | 41.59 | 29.97 |
| B | 40.17 | 100.0 | 18.52 | 23.65 | 22.22 | 27.92 | 25.93 | 37.89 | 41.31 | 29.06 |
| C | 30.36 | 29.02 | 100.0 | 26.79 | 29.46 | 25.45 | 17.86 | 30.80 | 33.48 | 27.68 |
| D | 31.50 | 32.68 | 23.62 | 100.0 | 32.68 | 28.35 | 23.62 | 36.61 | 39.76 | 31.10 |
| E | 29.00 | 29.00 | 24.54 | 30.86 | 100.0 | 23.42 | 18.22 | 31.23 | 37.55 | 24.91 |
| F | 33.44 | 30.06 | 17.48 | 22.09 | 19.33 | 100.0 | 25.15 | 30.37 | 30.98 | 24.23 |
| G | 33.33 | 33.70 | 14.81 | 22.22 | 18.15 | 30.37 | 100.0 | 35.93 | 38.89 | 25.56 |
| H | 30.13 | 29.69 | 15.40 | 20.76 | 18.75 | 22.10 | 21.65 | 100.0 | 38.39 | 22.10 |
| I | 28.63 | 30.53 | 15.79 | 21.26 | 21.26 | 21.26 | 22.11 | 36.21 | 100.0 | 24.63 |
| J | 27.45 | 28.57 | 17.37 | 22.13 | 18.77 | 22.13 | 19.33 | 27.73 | 32.77 | 100.0 |
| .6B:.3D:.1G | 30.74 | 57.87 | 15.31 | 37.28 | 22.39 | 24.50 | 29.16 | 32.63 | 34.99 | 24.74 |
| .5B:.3D:.2G | 31.50 | 53.50 | 15.94 | 37.37 | 21.79 | 25.91 | 33.62 | 34.63 | 38.38 | 25.67 |
| .4B:.3D:.3G | 30.97 | 49.42 | 14.85 | 38.50 | 20.05 | 23.31 | 35.79 | 33.00 | 35.61 | 24.36 |
| .33B:.33D:.33G | 31.89 | 47.88 | 16.62 | 39.70 | 22.44 | 26.26 | 37.41 | 33.75 | 34.54 | 25.28 |

Aitchison Distance Between Samples

A repeat of the three-sample mixture experiment was performed using samples A & B from the same family and the next most similar sample F. The performance of this mixture is consistent with our findings in the original experiment. The plots of this new simulation are shown below, including proportion estimation, stacked-bar plots, and error metric plots, for the evidences with various compositions (a-d). These plots are included in the Supplementary file as well.

We additionally considered how the estimator performs as the mixture proportion changes. We used a mixture of 2 samples and followed the performance of the estimator form 0% to 100%. As in our original experiment, when both samples are present, the addition of RAD makes a negligible difference in the estimated proportions.

This approach was repeated for a mixture of two samples with one of the two samples missing. In this scenario, RAD improves the estimation performance for the missing small to moderate proportions (the orange points in the plots below), particularly, for the RAD-FEAST.

3. The results go on to focus on how RAD might improve ST and FEAST via source removal when considering two and three source mixtures. How does RAD compare to simplistic post-processing source removal approaches? One very basic approach might be to simply remove sources from ST/FEAST output if their estimated proportion is <X%, set to a relatively small number such as X=1%, then redistribute the removed density to the leftover sources. This adds a user-selected threshold X, which RAD doesn’t require. RAD may still be useful as a parameterless approach. However, it would be greatly informative to understand how RAD compares to basic thresholding.

Thank you for your suggestion. We have included a comparison of RAD with ST and FEAST using a conservative threshold of 5%. The results of this new study agree with our original conclusion. For sets where all true sources are included, RAD does not make any improvement after the threshold cut-off (see the stacked-bar plots and error metric plots below, where a)-d) are for the evidences with various compositions).

However, for sets where a true source is missing, RAD leads to improvements in proportion estimates (see the stacked-bar plots and error metric plots below; a)-d) are for the evidences with various compositions), where the ST and FEAST methods assigned some proportions to the false samples (i.e., the pink and orange portions).

4. There does not appear to be a file representing the summary data used to generate the Figures publically available. There is also no source code that I could find at:

<https://cals.arizona.edu/~anling/software/RAD/>

The RAD program has been deposited in GitHub:

<https://github.com/anlingUA/RAD> in addition to an example dataset.

5. In part 2.1, hierarchical clustering is mentioned but it is unclear whether it was used for the simulation analysis.

This step relates to the “environment” composite used by SourceTracker and FEAST. When related samples are suspected, hierarchical clustering can bin similar samples to avoid within-environment comparisons. In our simulation, with or without the hierarchical clustering step does not affect the final result.

6. In part 2.2.2 with ST, it is not clear what the hyperparameters alpha and beta are set to after parameter tuning, or how they were tuned.

Hyperparameters were initially tuned using the default SourceTracker tuning function, but computation speeds were slow and parameter tuning showed minimal improvement. For the analyses displayed in our paper, we utilized default hyperparameters of 0.001. In the revision we have added more explanations.

**Reviewer 2**

1. Page 4, Lines 72-77: The authors stated as “We propose two new methods for estimating the source proportions based on the ratio of ecological similarity between sources and the sink sample. The proposed methods are evaluated and compared with Bayesian SourceTracker using comprehensive simulation studies”. ==> However, the authors proposed to use Aitchison difference to select important sources and then adapted it to three existing methods including naïve, SourceTracker, and FEAST approaches. Therefore, the authors proposed one method, then applied it into three existing methods/tools. Please clarify it and update in the manuscript.

Thank you for addressing this. RAD is essentially used as a preprocessing step for other algorithms. We have updated the manuscript to clarify it.

2. The simulated data sets have very simple proportions. The study has focused on scenarios where the sink is generated from a mixture of two to three sources. Does the proposed method work for many sources, not just two or three?

We have additionally included a mixture of four samples. For mixtures of four samples, the performance of RAD is consistent with our original conclusions. See the plots below.

3. Does the proposed method work for longitudinal data sets?

Incorporating information from multiple time points is not a simple extension to make and is outside the scope of our study. However, we have opted to investigate whether source estimates are consistent across time. This comparison looked at the performance using source samples from days 2-5 (still use the original hand sample experiment in Lax et al. 2015). For longer time, we expect degradation in prediction quality due to accumulating perturbations. Identifying a stable set of microbes will be necessary to gain a better prediction, which is outside the scope of this study.

For the performance on days 2-5, the estimated proportions are fairly consistent. See the plots below.

4. It is possible that the reviewer missed some important point from the manuscript. The original data set from Lax et al. ([13] in the manuscript) provide longitudinal data sets. Shenhav et al. ([15] in the manuscript) also provides many sources data set. So, the authors should provide more intensive data analysis using the data sets to validate the proposed method.

Thanks for your suggestion. In the revised paper we added a new real data analysis. Using an experiment in predicting fecal contamination in water from Brown et al. (2019) we have compared the performance of RAD to Bayesian Source-Tracker and FEAST. The data from this experiment was analyzed at the Genus level and was rarefied to the smallest library size of 9,000. When the sources included were lake water, sewage effluent, cow fecal matter, and horse fecal matter, RAD was correctly able to identify the true sources (see the plot below) where the evidence/sink contains (a) lake water and sewage effluent or (b) lake water and cow fecal matter. Particularly, for experiment in (b), both SourceTracker and FEAST detected two samples, effluent and horse, which were not in the evidence.

5.. Figures of the results only show the prediction proportions and error bars. Please consider to choose various visualization techniques for readers to be more readily interpretable of the benefit of the proposed methods.

Thank you for your input. We have opted to include stacked bar charts to offer a clearer visualization of the improvement in prediction, as well as the scatterplot for true vs predicted proportions. Lastly, we have also included four additional error measurements to provide a wider view of the method performance.

6. The data that the authors tested is available, but the reviewer cannot find any program scripts or software tools. Open-source package will help users to test and cite your work, so the reviewer strongly suggests to make it available in public.

The RAD program has been deposited in GitHub:

<https://github.com/anlingUA/RAD> in addition to an example dataset.
